# Canine mesenchymal stem cell-derived exosomes attenuate renal ischemia-reperfusion injury through miR-146a-regulated macrophage polarization

**DOI:** 10.3389/fvets.2024.1456855

**Published:** 2024-09-09

**Authors:** HaiFeng Liu, Hongchuan Deng, Haocheng Huang, Jiahui Cao, Xinmiao Wang, Ziyao Zhou, Zhijun Zhong, Dechun Chen, Guangneng Peng

**Affiliations:** ^1^Department of Veterinary Surgery, College of Veterinary Medicine, Sichuan Agricultural University, Chengdu, China; ^2^College of Animal and Veterinary Sciences, Southwest Minzu University, Chengdu, China

**Keywords:** mesenchymal stem cell, exosomes, ischemia-reperfusion, miR-146a, 3 macrophage polarization

## Abstract

**Introduction:**

The most common factor leading to renal failure or death is renal IR (ischemia-reperfusion). Studies have shown that mesenchymal stem cells (MSCs) and their exosomes have potential therapeutic effects for IR injury by inhibiting M1 macrophage polarization and inflammation. In this study, the protective effect and anti-inflammatory mechanism of adipose-derived mesenchymal stem cell-derived exosomes (ADMSC-Exos) after renal IR were investigated.

**Method:**

Initially, ADMSC-Exos were intravenously injected into IR experimental beagles, and the subsequent assessment focused on inflammatory damage and macrophage phenotype. Furthermore, an *in vitro* inflammatory model was established by inducing DH82 cells with LPS. The impact on inflammation and macrophage phenotype was then evaluated using ADMSC and regulatory miR-146a.

**Results:**

Following the administration of ADMSC-Exos in IR canines, a shift from M1 to M2 macrophage polarization was observed. Similarly, *in vitro* experiments demonstrated that ADMSC-Exos enhanced the transformation of LPS-induced macrophages from M1 to M2 type. Notably, the promotion of macrophage polarization by ADMSC-Exos was found to be attenuated upon the inhibition of miR-146a in ADMSC-Exos.

**Conclusion:**

These findings suggest that miR-146a plays a significant role in facilitating the transition of LPS-induced macrophages from M1 to M2 phenotype. As a result, the modulation of macrophage polarization by ADMSC-Exos is achieved via the encapsulation and conveyance of miR-146a, leading to diminished infiltration of inflammatory cells in renal tissue and mitigation of the inflammatory reaction following canine renal IR.

## Introduction

The injury of the kidney due to IR is a significant cause of renal disease in animals and humans, leading to severe morbidity and mortality (1). Acute IR injury results in a rapid decline in renal function, exacerbates kidney damage, and initiates a cascade of renal inflammation (2, 3). The occurrence of tissue inflammation is closely associated with the phenotype of macrophages, which are key components of the innate immune system and play a crucial role in regulating inflammation following renal reperfusion (4). After reperfusion, M1 macrophages create a pro-inflammatory environment and aid in the clearance of cellular debris, followed by an increased prevalence of M2 macrophages. In addition to secreting anti-inflammatory cytokines, M2 macrophages produce various growth factors that promote collagen synthesis and cell proliferation (5), thus playing a crucial role in the process of inflammation (6). The imbalance between M1 and M2 macrophages presents an opportunity for potential treatment targeting for renal IR injury (7, 8).

ADMSCs are stem cells known for their potential to differentiate into various cell types, making them suitable for tissue repair and exerting anti-inflammatory, anti-apoptotic, and pro-angiogenic effects (9). Studies have demonstrated that ADMSCs have the ability to mitigate pro-inflammatory cytokines produced by macrophages and promote macrophage polarization toward the anti-inflammatory M2 phenotype, both *in vivo* and *in vitro* (10, 11). The advantages of ADMSCs, including low invasiveness, high proliferation rates, and easy availability via minimally invasive surgery, make them promising for future clinical applications (12). However, the underlying mechanism of ADMSCs remains unclear, and growing evidence suggests that the beneficial effects of stem cells in renal injury models may be attributed to their paracrine signaling mechanisms rather than their differentiation ability (13–16).

Stem cell-derived exosomes, which are lipid bilayer-bound vesicles containing various bioactive molecules (proteins, nucleic acids, lipids, and RNA molecules), have been shown to play a crucial role in the biological effects of ADMSCs (17). Recent studies have demonstrated that exosomes derived from ADMSCs have the ability to promote recovery in animal models of tissue injury and inflammatory diseases, thereby improving the prognosis for IR injury (18, 19). However, whether ADMSC-derived exosomes can stimulate macrophages to create an anti-inflammatory environment in renal IR injury remains unknown.

MiRNAs are crucial component of Exos, and studies have found that many miRNAs in stem cells can be packaged and transported through Exos playing a vital role in immune regulation. For instance, miR-125b-5p in MSC-Exos may promote renal tubular repair and improve renal IR damage (20); miRNA-199-5p may alter macrophage polarization and enhance renal epithelial endoplasmic reticulum stress to alleviate renal damage (21). MSC-Exos inhibits atherosclerosis by deliver miR-145 (22). Furthermore, a considerable number of ADMSC-Exos-coated miRNAs have important immunomodulatory properties, such as miR-23a, miR-223, miR-146a, and miR-181b, etc. (23–27). According to literature reports, miR-23a and miR-146a are highly enriched in ADMSCs, and both have the ability to regulate macrophage phenotype and inflammation (28, 29). In our study, we established a model of renal IR injury in canines and intervened with ADMSC-derived exosomes. The study revealed that ADMSC-derived exosomes regulated the inflammatory environment in the renal IR model and facilitated macrophage polarization by transporting miR-146a, conferring protection against renal IR injury in canines.

## Materials and methods

### Isolation of ADMSC-Exos

Isolation, culture, extraction and purification of canine ADMSC and ADMSC-Exos was according to the separation method previously (30). Briefly, ADMSCs were isolated from inguinal fat tissue and cultured in complete culture medium (LG-DMEM+10% FBS) supplemented with 10% EQ-fetal bovine serum at 37°C and 5% CO_2_ following digestion with type I collagenase, filtration, and centrifugation. The supernatants of P3-P4 ADMSCs were ultracentrifuged (Thermo Scientific^™^ Sorvall^™^ WX 80 +, United States) in order to isolate exos. A concentrated solution in supernatants was enriched using sucrose density gradient centrifugation. An ultracentrifugation tube was layered with sucrose solution and concentrated solution at a 1:8 ratio, and centrifuged at 110,000 g for 70 min at 4°C to collect heavy water layer. PBS was added to resuspend the precipitate, which was centrifuged at 110,000 g at 4°C for another 70 min. The resuspended precipitate was resuspended in 50–100 μL of PBS.

### Animals model

Nine male beagles aged 3 years were randomly assigned to blank (left kidney exposure without renal vessel clamping and right nephrectomy, *n* = 3), model (left renal vessel clamping and right nephrectomy, *n* = 3), and treatment groups (IR model with administration of Exos, *n* = 3). In the treatment group, the kidney was exposed surgically and Exos (180 ug/kg) dissolved in PBS was injected into the renal cortex with a 1 mL syringe after the clamp was removed. Beagles were maintained under a circadian rhythm with *ad libitum* access to food and water. The beagles were euthanized after the experiment. A Guide for the Care and Use of Laboratory Animals published by the National Academy Press was used to care for and handle the dogs (National Institutes of Health Publication No. 85-23, 1996 revision). Institutional Animal Care and Use Committee approval was obtained for this study at Sichuan Agricultural University (No. SYXK-2019-187).

Immediately after adaptive feeding (1 week), nine beagles were examined physically, had routine blood tests, and had kidney B-ultrasound screenings performed. The inter-group comparison did not reveal any statistically significant difference in terms of body weight.

In all experimental dogs, isoflurane was inhaled after zolazepam, tiletamine, and propofol were administered as anesthesia inductions. In order to induce chemical injury in the left renal vessels, a ventral midline incision was made and a noninvasive hemostatic clip (5.7 cm, Shanghai Medical Instrument Co. Ltd., China) was used to clip the vessels for 60 min, therefore, reperfusion injury was caused by releasing the clip. Immediately after releasing the clip, a right nephrectomy was performed. The abdominal cavity was rinsed with physiological saline, and the incision was routinely sutured.

The serum samples were collected before surgery and 30 h after reperfusion for further analysis. The kidney tissues were collected by laparotomy after 30 h of reperfusion and stored at −80°C. The same trained surgeons performed all surgical procedures in the same environment using sterile instruments.

### HE staining

A section of kidney tissue was obtained from the caudal pole of the left kidney, fixed in 10% buffered formalin, embedded in paraffin, and slice into 3 μm thick sections.

Each sample was stained with HE to evaluate histopathological changes. Pathological section images of each sample were analyzed to check for infiltration of inflammatory cells after surgery. For inflammatory cell infiltration analysis, we randomly selected 10 fields (400× magnification) in each kidney section. The percentage of inflammatory cells is assessed by counting the number of nuclei in inflammatory cells and comparing them with the total number of nuclei in the same field of view (31).

### DH82 cell culture

Canine macrophage line DH82 (Cell Center, Shanghai Academy of Biological Sciences, Shanghai, China) was cultured in MEM medium (Solarbio, China) supplemented with 1% penicillin–streptomycin and 10% FBS (Bioindustries, Israel). Cells were maintained at 37°C with 5% CO2 in a humidified environment. A fresh medium containing 200 ng/mL LPS (Sigma-Aldrich, United States) was added to the DH82 cells for 24 h to induce the M1 phenotype.

### Fluorescent labeling of exosomes: endocytosis experiments

The collected exosomes were labeled with Dil (Solarbio, China) according to the manufacturer’s protocol, then washed and re-suspended in PBS. DH82 was inoculated in a petri dish and co-cultured with Dil-labeled exosomes for 24 h, and exosomes were replaced by exosome-free medium (LG-DMEM +10% Exosome-free FBS) and PBS as controls, respectively. Then, the cells were washed three times with PBS and fixed in 4% paraformaldehyde. The nuclei were stained with DAPI and the images were taken by spectroscopic laser confocal microscopy (Olympus, Japan).

### Transfection of miRNA-146a

The Gene ID of the miRNA-146a is 100,885,925, and the and the sequences is tgagaactgaattccatgggtt. DH82 cells were collected after 24 h stimulation with 200 ng/mL LPS (Sigma Aldrich). According to the manufacturer’s method, in the Opti-MEM medium, miR-146a mimics (Shanghai Gene Pharmaceutical Co., LTD., Ltd., China) or corresponding control oligonucleotides (miR-146a mimic NC) were transfected into macrophages by Lipofectamine 2000 transfection reagent (Thermo Fisher, United States). Mimic is to mimic natural double strand of miRNA cut by Dicer enzyme, which increases miRNA content after instantaneous transfer into the cell. After 12 h of culture, the Opti-MEM medium was replaced with a complete medium, and samples were collected 48 h after transfection. Using the same method, canine ADMSCs were transfected with a 200 pmol/ well miR146 inhibitor (Shanghai Gene Pharmaceutical Co., Ltd., China) or corresponding control oligonucleotide (miR-146a inhibitor NC). Inhibitor is a single chain that is fully complementary to the corresponding miRNA. After transient transfer into the cell, it can release the inhibition of the target gene. After incubation for 12 h, the Opti-MEM medium transform to exosome-free medium. After 24 h, the cell supernatant was collected and exosomes were extracted. Exosomes were co-cultured with LPS-stimulated DH82 cells (The final concentration of exosomes was 50 μg / mL, cell confluency 60%), and cells and cell supernatants were collected 48 h later for follow-up experiments (each group of inhibitors will be treated with Exosomes). All oligomers are synthesized by GenePharma (Shanghai, China).

### ELISA

Canine ELISA kits (R&D Systems, Inc., Minneapolis, MN, United States) were used to detect serum concentrations of interleukin IL-1β, IL-10, IL-6, TNF-α, and TGF-β in the previously collected Serum, kidney, and the DH82 cell supernatant samples, and the concentrations of iNOS and Arg-1 in renal tissue and DH82 cells.

### qPCR

For gene expression analysis, the renal tissue or cells were harvested and lysed in RNAiso plus (Solarbio, China), after which RNA was quantified with NanoDropOneC (Thermo Scientific, United States) and reverse-transcribed using the PrimeScript^™^ RT Reagent Kit (Solarbio, China) to obtain cDNA (add 1–5 ug RNA to the kit). For miR-146a expression analysis, after RNA isolation, first-strand cDNA was synthesized via a miRNA First Strand Synthesis Kit (TransGen Biotech, China) according to the manufacturer’s instructions. qPCR was performed in triplicate using the SYBR Premix Ex Taq on the ABI ViiA7Dx real-time PCR system. qPCR was performed in triplicate using the Bio-Rad real-time PCR system (CFXConnect, United States). The expression levels of mRNA were calculated according to the comparative threshold cycle value (2^−ΔΔCt^) method and were normalized against GADPH or RNT43. The primers for target genes are included in Table 1.

**Table 1:** mRNA primer sequences.

Gene	Primer sequence (5′-3′) sense	Primer sequence (5′-3′) anti-sense
Canis IL-1β	CCACCCTACAGCTAGAGAAGGT	CGACTTGAGAGGTGCTGATG
Canis IL-6	GCACTGAGAAAGGAGATGTGTG	TCCCTCCAGTTTGGGAAGATG
Canis TNF-α	CTCCCAAATGGCCTCCAACT	CAGCTTCGGGGTTTGCTACA
Canis iNOS	AGCCTCATATCTATCTCCCC	ATCAAGGAGTCATACAGGGA
Canis IL-10	ACCCAGGATGGCAACTCTTC	TGGTCGGCTCTCCTACATCT
Canis TGF-β	CATGGCATGAACCGACCCTTCC	CCGTGGAGCTGAAGCAGTAGTTG
Canis Arg-1	AATGGAAGTGAACCCATCTC	TGAACAACCTGGGAATATGG
Canis GADPH	CCCTGAGCTGAACGGGAAG	CTCCGATGCCTGCTTCACT
Canis miR-146a	CCGTGAGAACTGAATTCCATGGGTT	
Canis miR-23a	CGGATCACATTGCCAGGGATTT	
RNT43	CTTATTGACGGGCGGACAGAAAC	

### Statistical analysis

GraphPad Prism 7.0 (GraphPad Software, United States) was employed to perform statistical analyses. Data are expressed as the mean ± SD. Statistical differences were evaluated using Student’s *t*-test or one-way ANOVA with Tukey’s *post hoc* test for multigroup comparisons, and *p* values < 0.05 were considered significant.

## Results

### ADMSC-Exos reduces inflammatory response after renal IR injury and promotes macrophage transformation to M2-like phenotype

**Figure 1:**
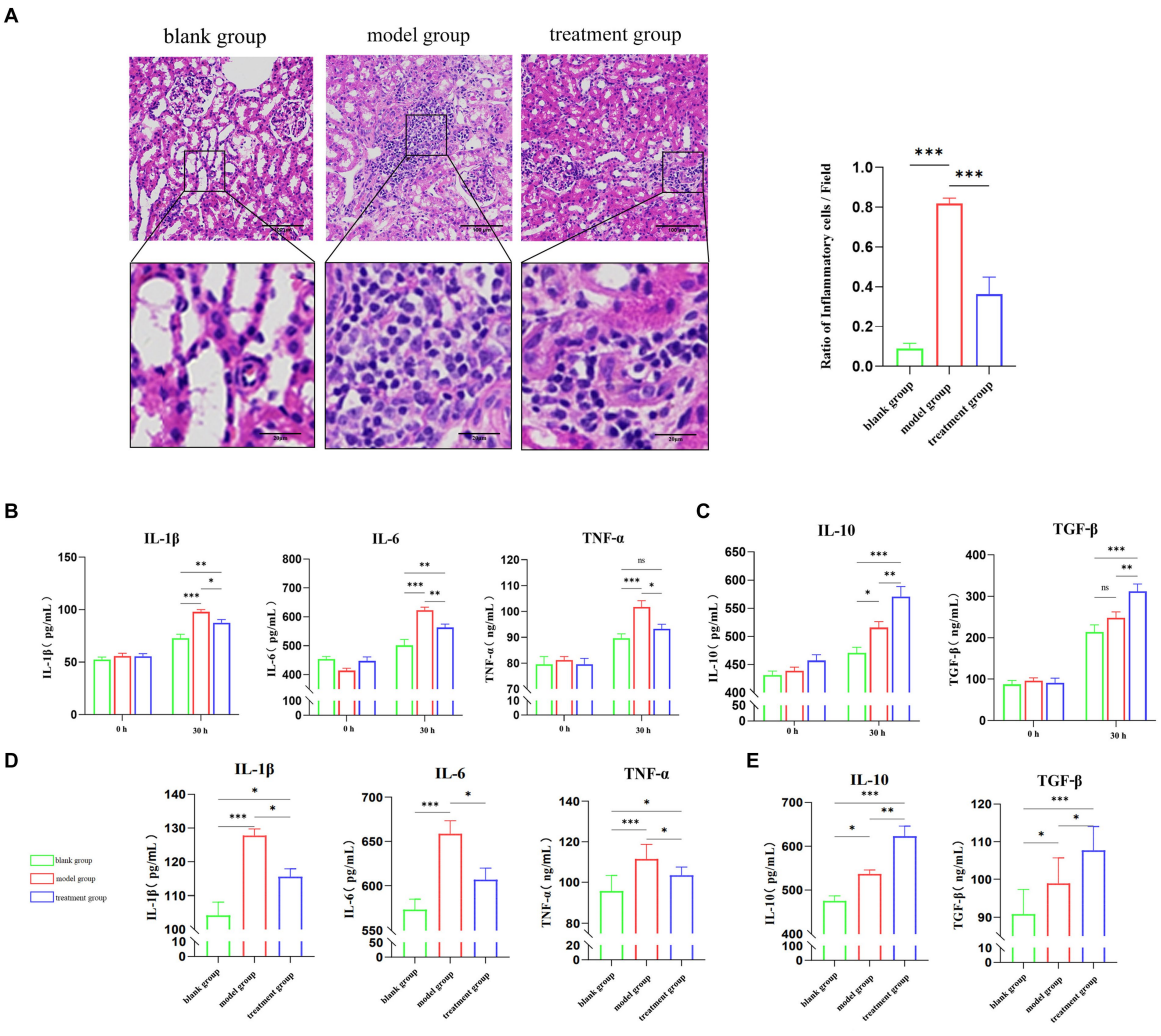
ADMSC-Exos reduces inflammatory response and improve renal injury after renal IR injury. **(A)** HE staining and inflammatory cell infiltration within the ischaemic reanl 30 h following operation (*n* = 3). Scale bar = 100 μm. **(B)** Contents of pro-inflammatory cytokines IL-1β, IL-6 and TNF-α in the serum of experimental dogs through ELISA in each group 30 h following operation (*n* = 3). **(C)** The levels of anti-inflammatory cytokines IL-10 and TGF-β in the serum of experimental dogs through ELISA in each group 30 h following operation (*n* = 3). **(D)** Contents of pro-inflammatory cytokines IL-1β, IL-6 and TNF-α in the kidney tissue of experimental dogs through ELISA in each group (*n* = 3). **(E)** The contents of anti-inflammatory cytokines IL-10 and TGF-β through ELISA in the kidney tissue of experimental dogs in each group (*n* = 3). *Difference compared in different groups, *p* < 0.05; **Significant difference compared in different groups, *p* < 0.01; ***Extremely significant difference compared in different groups, *p* < 0.001.

In order to investigate ADMSC-Exos’ potential effects on macrophages, we examined inflammatory cell infiltration in the kidney following ADMSC-Exos administration. The left kidney tissue was collected 30 h after surgery, and we can observe that there are basically no pathological changes in the blank group, while there are a large number of inflammatory cells infiltration, tubular epithelial cell necrosis, and nuclear fragmentation in the model group. However, dogs treated with ADMSC-Exos showed significantly less inflammatory cell infiltration and smaller lesion area than those in the model group. These results suggest that ADMSC-Exos can alleviate tissue damage by reducing the infiltration of inflammatory cells after renal IR (Figure 1A).

ELISA was used to detect inflammatory factors in serum. The results showed that after kidney IR 30 h, the contents of serum IL-1β, IL-6, and TNF-α (tumor necrosis factor-α) were significantly reduced in the treatment group compared with the model group, and there was no significant difference in TNF-α between the treatment group and blank group (Figure 1B). Meanwhile, the contents of serum anti-inflammatory factors IL-10 and TGF-β (transforming growth factor-β) in the treatment group were significantly higher than those in the model group (Figure 1C). Inflammatory factors in renal tissues were also shown, and the content of pro-inflammatory factors was significantly lower than that of the model group after the intervention of ADMSC-Exos, and the content of anti-inflammatory factors was significantly higher than that of the model group (Figures 1D,E). The contents of inflammatory factors in serum and kidney tissues of experimental dogs in each group showed the same trend, suggesting that ADMSC-Exos could alleviate the inflammatory response of IRI to the kidney, reduce the content of pro-inflammatory cytokines and increase the release of anti-inflammatory factors.

**Figure 2:**
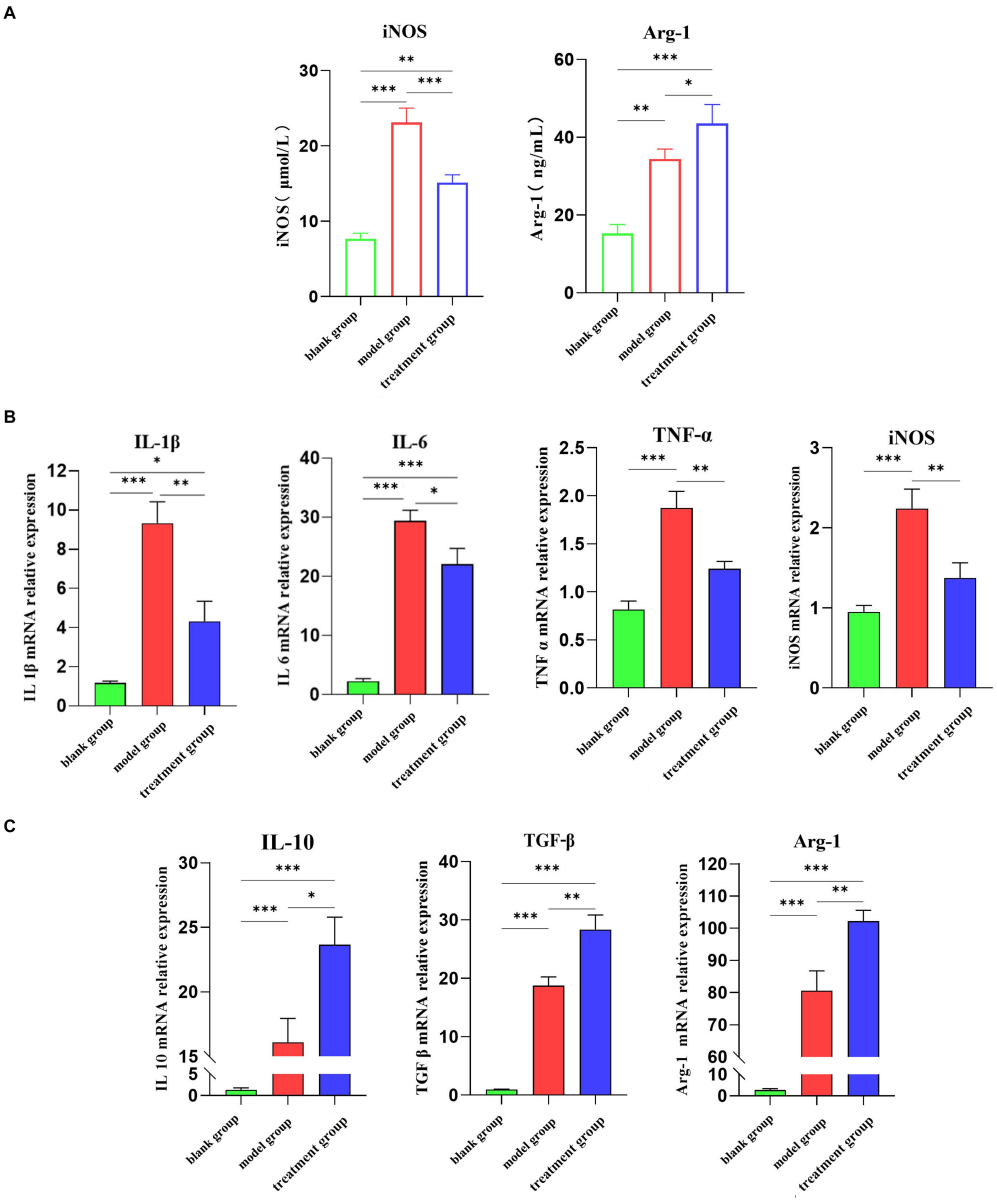
ADMSC-Exos promotes macrophage transformation to M2-like phenotype after renal IR injury. **(A)** Contents of M1 and M2 biomarkers via ELSIA in ADMC-Exo treatment of renal IR injury 30 h following operation. **(B)** Gene expression of pro-inflammatory cytokines IL-1β, IL-6 and TNF-α via qPCR in ADMC-Exo treatment of renal IR injury through ELISA in each group 30 h following operation (*n* = 3). **(C)** Gene expression of anti-inflammatory cytokines IL-10 and TGF-β via qPCR in ADMC-Exo treatment of renal IR injury through ELISA in each group 30 h following operation (*n* = 3). Difference compared in different groups, *p* < 0.05; **Significant difference compared in different groups, *p* < 0.01; ***Extremely significant difference compared in different groups, *p* < 0.001.

Firstly, the expression levels of M1-type macrophage marker iNOS (inducible nitric oxide synthase) and M2-type macrophage marker Arg-1 in the kidney were detected by ELISA (Figure 2A). Compared with the treatment group, the expression levels of M1-type macrophage marker (iNOS) in dogs treated with ADMSC-Exos were significantly reduced. At the same time, the expression level of the M2 macrophage marker (Arg-1, Arginase 1) increased after ADMSC-Exos treatment. In addition, qPCR analysis of M1 and M2 gene expression also showed a significant decrease in M1 markers (iNOS, IL-1b, IL-6, and TNF-α) and an increase in M2 markers (Arg1, IL-10, and TGF-β) (Figures 2B,C). In summary, these data suggest that MSC-Exos polarize macrophages away from the M1 phenotype and toward an M2-like state during renal IR injury.

### ADMSC-Exos converted inflammatory macrophages to M2 phenotype *in vitro*

**Figure 3:**
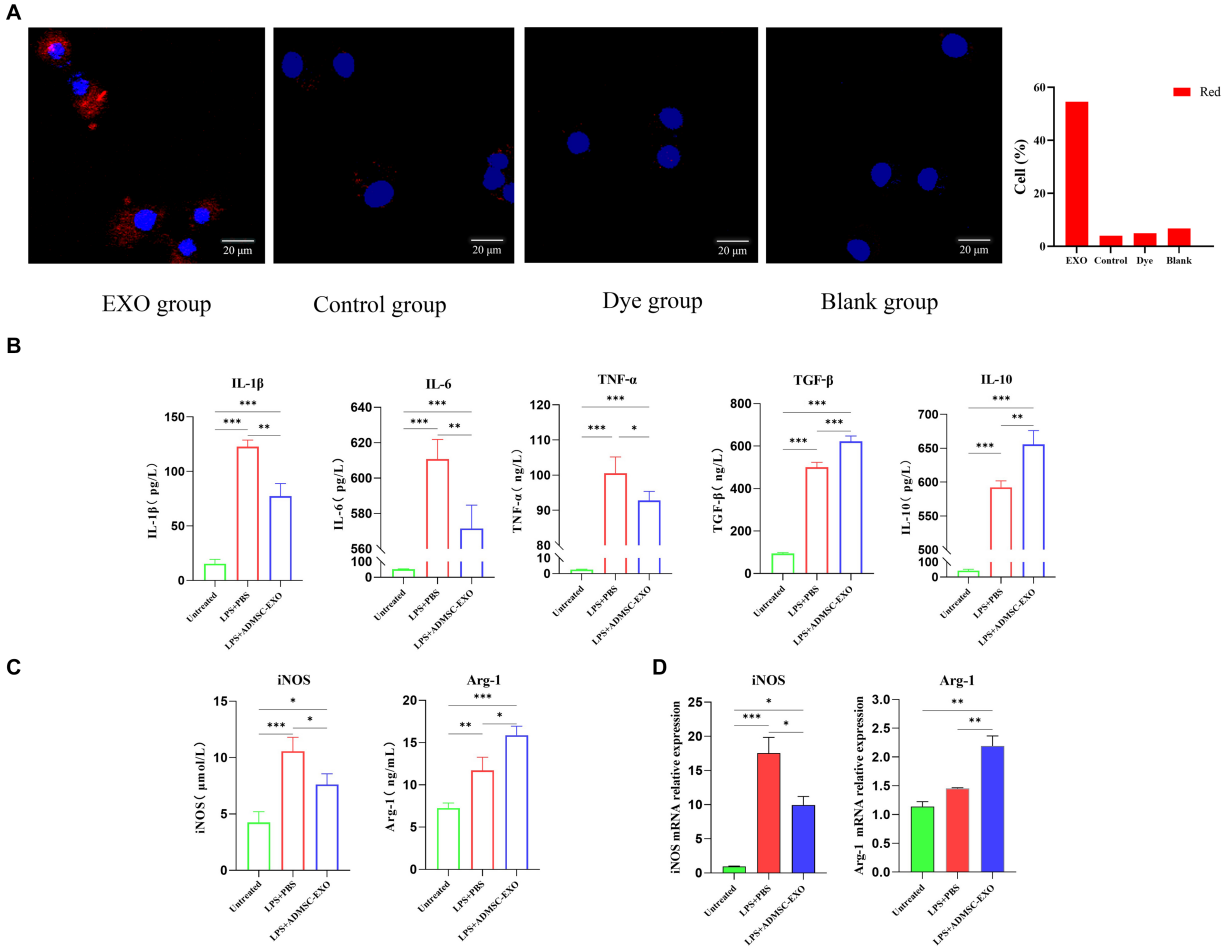
ADMSC-Exos converted inflammatory macrophages to M2 phenotype *in vitro*. **(A)** Immunofluorescence shows that Exo is captured by macrophages. **(B)** Contents of IL-1β, IL-6, TNF-α, IL-10, and TGF-β in cell supernatant after co-culture of ADMSC-Exos and macrophages. **(C)** Expression of M1 and M2 macrophage markers in each group via ELISA. **(D)** Gene expression of M1 and M2 macrophage markers in each group via qPCR. *Difference compared in different groups, *p* < 0.05; **Significant difference compared in different groups, *p* < 0.01; ***Extremely significant difference compared in different groups, *p* < 0.001.

*In vitro* experiments, ADMSC-Exos was labeled with Dil, and macrophages were co-cultured with ADMSC-Exos labeled with Dil (red) to observe uptake. Since Dil is a lipophilic membrane dye, to eliminate the interference of Dil non-specific staining, we set up a DiL-stained exosome-free serum medium for comparison with PBS. The results showed that ADMSC-Exos could be taken up in large quantities after 24 h co-culture with macrophages (Figure 3A).

For obtaining macrophages with M1 phenotypes, DH82 cells were stimulated with 200 ng/mL LPS (Sigma-Aldrich) for 24 h. Macrophages were pretreated with ADMSC-Exos (200 μg/mL) and the same amount of PBS was added as control, and samples were harvested 24 h later.

We observed the levels of IL-1β, IL-6, TNF-α, IL-10, and TGF-β in the culture supernatants, and assessed the expression of iNOS and Arg1 in macrophages. The ADMSC-Exos were discovered to hinder the production of IL-1β, IL-6, and TNF-α induced by LPS, while facilitating the increase in IL-10 and TGF-β (Figure 3B). ELISA and qPCR results also showed that M2-type macrophage marker (Arg-1) were levels increased significantly after ADMSC-Exos treatment, and M1 macrophage marker levels decreased (iNOS) (Figures 3C,D). All together, these results indicated that MSC-Exos facilitated macrophage polarization toward M2 rather than M1.

### ADMSC-Exos promote M1-to-M2 macrophage polarization through miR-146a

**Figure 4:**
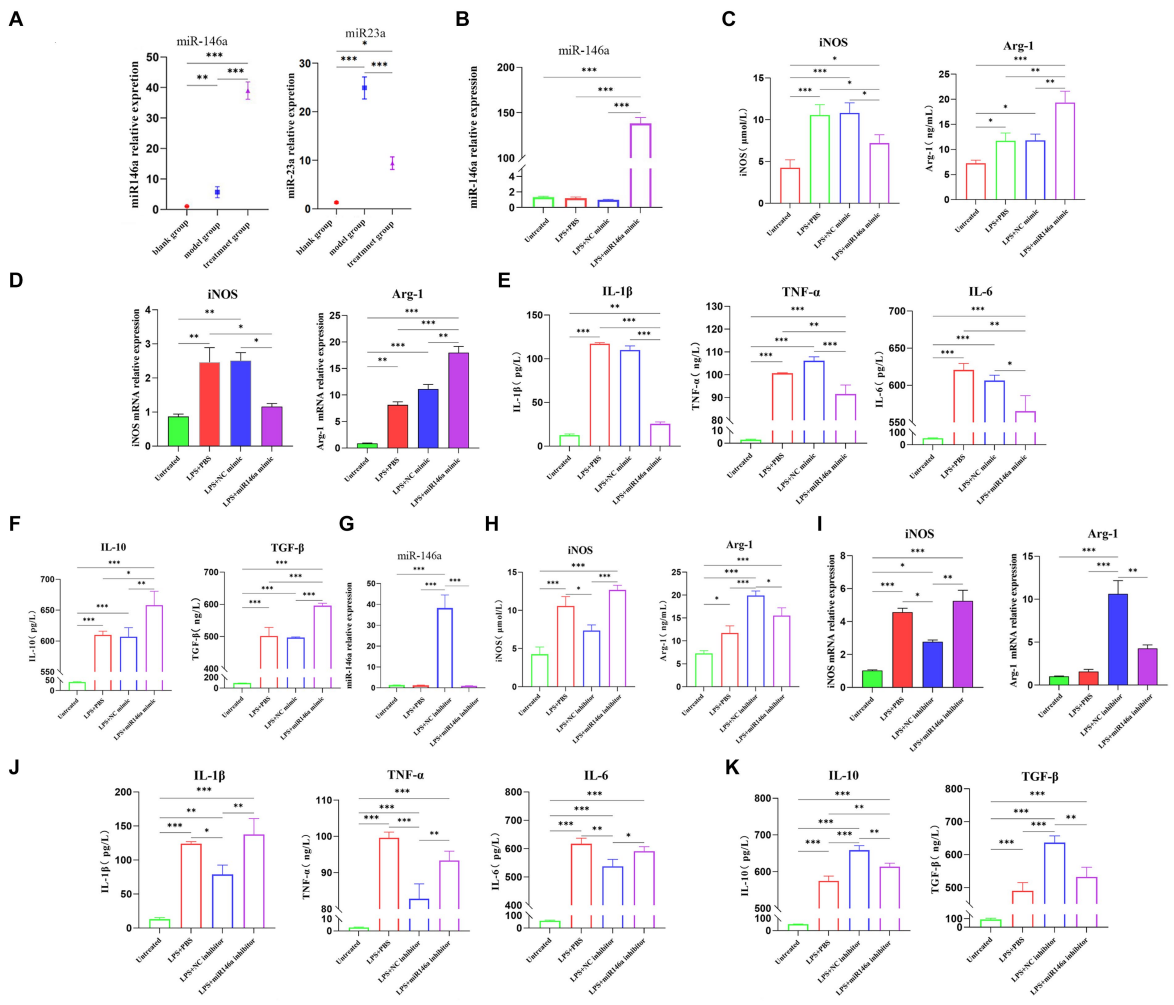
ADSC-Exo promote M1-to-M2 macrophage polarization through miR-146a. **(A)** The expression of miR-146a and miR-23a in renal tissues of experimental dogs was assessed after 30 h of renal ischemia-reperfusion. **(B)** qPCR analysis revealed a significant increase in the content of miR-146a in the LPS + miR146a group following transfection of macrophages stimulated by lipopolysaccharide (LPS) with a miR-146a mimic. **(C)** Gene expression of M1 and M2 biomarkers of macrophages after transfection with miR146a mimic via qPCR. **(D)** Contents of anti-inflammatory factors in cell supernatant after transfection with miR-146a mimic. **(E)** Expression of M1 and M2 biomarkers of macrophages after transfection with miR146a mimic via ELISA. **(F)** Exos were isolated from ADMCS transfected with miR-146a inhibitor and NC inhibitor, and the content of miR-146a was quantified using qPCR. **(G)** The levels of pro-inflammatory factors in the cell supernatant were measured after transfection with miR-146a mimic. **(H)** Expression of M1 and M2 biomarkers of macrophages after transfection with miR146a inhibitor via ELISA. **(I)** Gene expression of M1 and M2 biomarkers of macrophages after transfection with miR146a inhibitor via qPCR. **(J)** Contents of pro-inflammatory factors in cell supernatant after transfection with miR-146a inhibitor. **(K)** Contents of anti-inflammatory factors in cell supernatant after transfection with miR-146a inhibitor. *Difference compared in different groups, *p* < 0.05; **Significant difference compared in different groups, *p* < 0.01; ***Extremely significant difference compared in different groups, *p* < 0.001.

We detected miRNA in canine kidney tissues of the *in vivo* experimental part and used qPCR to detect the expression levels of miR-146a in the kidney tissues of each group. The results showed that after the intervention of ADMSC-Exos in the canine renal IRI model, the expression level of miR-146a in renal tissue was significantly higher than that in the other two groups, and the changing trend was consistent with the trend of M2-type macrophage markers (Figure 4A).

Next, step was to investigate the direct effects of miR-146a on macrophages. DH82 cells transfected with a correspondent mimic of miR-146a displayed higher levels of miR-146a after being transfected (Figure 4B). The cells collected 48 h after transfection were detected by ELISA and qPCR. The content of iNOS of M1 biomarkers in the LPS + miR-146a mimic group was lower than that of the LPS and LPS + NC (negative control) mimic group, and the expression of M2 biomarker Arg-1 was significantly higher than that of the other two groups (Figure 4C). At the gene level, the relative quantitative results of qPCR also showed the same trend (Figure 4D). The inflammatory factors in the supernatant were detected by ELISA (Enzyme-linked immunosorbent assay). The results also showed that the contents of pro-inflammatory factors IL-1β, IL-6, and TNF-α in the supernatant were significantly decreased after transfection of miR-146a mimic (Figure 4E), while the contents of anti-inflammatory factors IL-10 and TGF-β were significantly increased (Figure 4F). In conclusion, miR-146a would induce the transformation of macrophages from M1 type to M2 type after LPS stimulation.

In order to verify the involvement of miR-146a in ADMSC-Exos, we suppressed the activity of miR-146a in ADMSC-Exos, by introducing a miR-146a inhibitor into ADMSCs and subsequently extracted the ADMSC-Exos from the culture supernatants. MSC-Exos showed significantly lower levels of miR-146a in comparison with NC inhibitor MSC-Exos based on qPCR analysis (Figure 4G). After treating LPS-stimulated macrophages with NC inhibitor MSC-Exos or miR-146a inhibitor MSC-Exos for 48 h, the cells and supernatant were collected for ELISA and qPCR analysis. There was a significant reduction in iNOS expression in the LPS + NC inhibitor group versus the LPS + miR-146a inhibitor group. However, the expression level of M2-type marker Arg-1 showed the opposite trend, and the regulation effect of miR-146a inhibitor ADMSC-Exos on macrophage phenotype was almost lost (Figure 4H). qPCR showed the same results, although miR-146a inhibitor ADMSC-Exos could still up-regulate the expression of Arg-1, its regulatory capacity was much lower than that of NC inhibitor ADMSC-Exos (Figure 4I). The content of inflammatory cytokines in the cell supernatant was also clear: the content of inflammatory cytokines secreted by macrophages after co-culture with miR-146a inhibitor ADMSC-Exos was consistent with that of macrophages after LPS induction only. However, macrophages co-cultured with NC inhibitor ADMSC-Exos still had a strong regulatory ability on the secretion of inflammatory factors (Figures 4J,K). It is suggested that miR-146a plays an important role in the polarization of macrophages mediated by ADMSC-Exos.

## Discussion

In our previous studies, the canine ADMSCs were successfully isolated and cultured from canine subcutaneous adipose tissue. Meanwhile, the Exos was extracted from ADMSCs via using sucrose-heavy water density layer ultra-centrifugation. After ADMSC-Exos were used to intervene in the canine renal IR model, the degree of renal oxidative stress, apoptosis rate of renal cortical cells, and renal IR damage were reduced. Therefore, the renal tissue structure was effectively protected (30).

Renal IR is one of the most common renal injuries which can be caused by various prerenal pathogenic factors and surgical procedures that require temporary occlusion or reduction of blood supply to the kidney (32). After renal IR, various damage-related molecules were released by damaged tubular epithelial cells (33), which activated innate immunity (34). Macrophages and neutrophils are the main infiltrating cells in renal IR-induced inflammation. After injury, macrophages will recruit neutrophils to the inflammatory region, while neutrophils will recruit inflammatory monocytes, thereby initiating the inflammatory cascade (35). Furthermore, as key cells in inflammatory response and tissue homeostasis, macrophages play different roles in various stages of inflammatory response by altering their phenotype. Previous studies have demonstrated that ADMSCs can reduce the inflammatory response. However, the specific mechanism by which ADMSCs affect the dissipation of the inflammatory response remains unclear. Therefore, the immunomodulatory effect of MSCs on canine renal IR injury needed to be explored.

In this work, the experimental results verified that macrophages’ polarization was effectively altered by ADMSC-Exos, which made macrophages more biased toward M2 rather than M1 phenotype. Thus, the inflammatory cascade was reduced, enhancing subsequent repair activity. In addition, macrophage polarization and inflammatory cytokine secretion were regulated by ADMSC-Exos by transporting miR-146a.

Studies have shown that stem cells exert a therapeutic effect in models of acute and chronic kidney injury, primarily through their paracrine exosomes. The researchers found that Exos derived from MSCs showed an extremely high efficacy to treat tissue damage and immune regulation in a variety of animals, suggesting that exogenous MSC-Exos could effectively ameliorate renal IR damage through a renal IR model (18, 36). Xia et al. found that ADMSC-Exos alleviated ALI in mice by regulating the phenotype of macrophages in a mouse model of LPS-induced acute lung injury (ALI) (37).

The experimental results suggest that ADMSC-Exos can significantly reduce inflammatory cell infiltration following renal IR, alter the levels of inflammatory factors (IL-6, TNF-α, etc.) in renal tissue and serum, promote the transformation of macrophage phenotype from M1 to M2 type, and alleviate the inflammatory response. Furthermore, the co-culture of ADMSC-Exos with the canine macrophage line DH82 *in vitro* provides further evidence to support this conclusion.

MiR-23a and miR-146a are highly enriched in ADMSCs, and both have the ability to regulate macrophage phenotype and inflammation. By detecting the kidney tissue after ADMSC-Exos intervention in canine renal IR, it was found that miR-146a/miR-23a expression levels were consistent with the expression levels of M2-type/M1-type markers in macrophages, respectively. Previous research has demonstrated that the introduction of miRNA-146A-5P-modified MSCs can effectively prevent diabetic nephropathy in rats by promoting macrophage polarization toward M2 type (38). Our experiments also focused on miR-146a’s promotion of M2-type macrophages to reduce the inflammation. So miR-146a was identified as our preferred research object. We then transfected miR-146a *in vitro* and found that the expression of M2-type macrophage markers was significantly increased, and expression of anti-inflammatory factors was also significantly higher than that of pro-inflammatory factors after transfecting miR-146a mimics macrophages in an inflammatory environment. On the contrary, when miR-146a content in Exos was inhibited, Exo’s regulatory capacity for macrophage transformation was significantly weakened. MiR-146a plays a crucial role in regulating various biological processes *in vivo*, such as the regulation of tissue inflammatory damage, cell apoptosis, and cell aging through the NAD/SIRT pathway (39–41). Its regulatory effect on macrophages involves the modulation of Notch1, TRAF1, NF-κB, TLR2, and other signaling pathways (22, 42, 43). And, our results demonstrate that miRNA-146a regulates macrophage polarization to reduce inflammatory reaction.

Renal IR will cause renal inflammation and renal function damage, thus affecting the normal physiological function of animals. ADMSC-Exos treatment can reduce renal inflammatory response, avoid renal inflammatory damage and even fibrosis caused by long-term IR, so as to ensure the overall health of the animal. This study elucidates that canine ADMSCs-derived Exos-miR-146a can mitigate inflammatory reactions in IR injury by inducing the polarization of macrophages toward the M2 phenotype. These findings lay the groundwork for further investigations into the specific impacts of various miRNAs on macrophage function. Simultaneously, this study represents a unique utilization of miR-146a derived from ADMSC-Exos in canines, offering a foundational framework for potential clinical interventions in renal conditions, particularly IR injury. However, there are still some problems to be solved here. During the experiment, we found that even if the content of miR-146a in ADMSC-Exo was reduced, ADMSC-Exo still had a weak ability to regulate the polarization of macrophages. It is suggested that the regulation of macrophage polarization mediated by ADMSC-Exo does not solely depend on miR-146a, and other signaling molecules contained in ADMSC-Exo may also have the ability to regulate macrophages, but we have not conducted more studies on this, and the exploration of specific types and functions of other signaling molecules can be regarded as the direction of future research. In clinical Settings, ADMSC-Exos is usually treated by intravenous injection, so there are problems such as scattered therapeutic effect and poor targeting function, so improving the targeted therapeutic effect of ADMSC-Exos is also a future direction of research.

## Conclusion

In conclusion, our experimental results show that ADMSC-Exos can reduce the inflammatory response after canine renal IR and that miR-146a is enriched in extracted exosomes, which can inhibit inflammation and promote the polarization of M1 macrophages to M2 macrophages. With low immunogenicity and relatively easy access, exosomes have broad applications compared to cell therapy. By exploring the mechanism of Exos-miRNA, we can clarify the different functions of various miRNAs in Exos and improve the application range of Exos. In the clinical treatment of canine, there is a lack of effective treatment for IR. Refining the investigation into the impact and mechanism of exosome therapy presents a novel approach for addressing renal IR injury, and it also a novel research idea for addressing other tissue inflammatory injuries.

## Data Availability

The original contributions presented in the study are included in the article/Supplementary material, further inquiries can be directed to the corresponding author.
