# Supplementary figures and images for: Canine mesenchymal stem cell-derived exosomes attenuate renal ischemia-reperfusion injury through miR-146a-regulated macrophage polarization
Source: Front Vet Sci. 2024 Sep 9;11:1456855. doi: 10.3389/fvets.2024.1456855 (PMC11417097; doi:10.3389/fvets.2024.1456855)

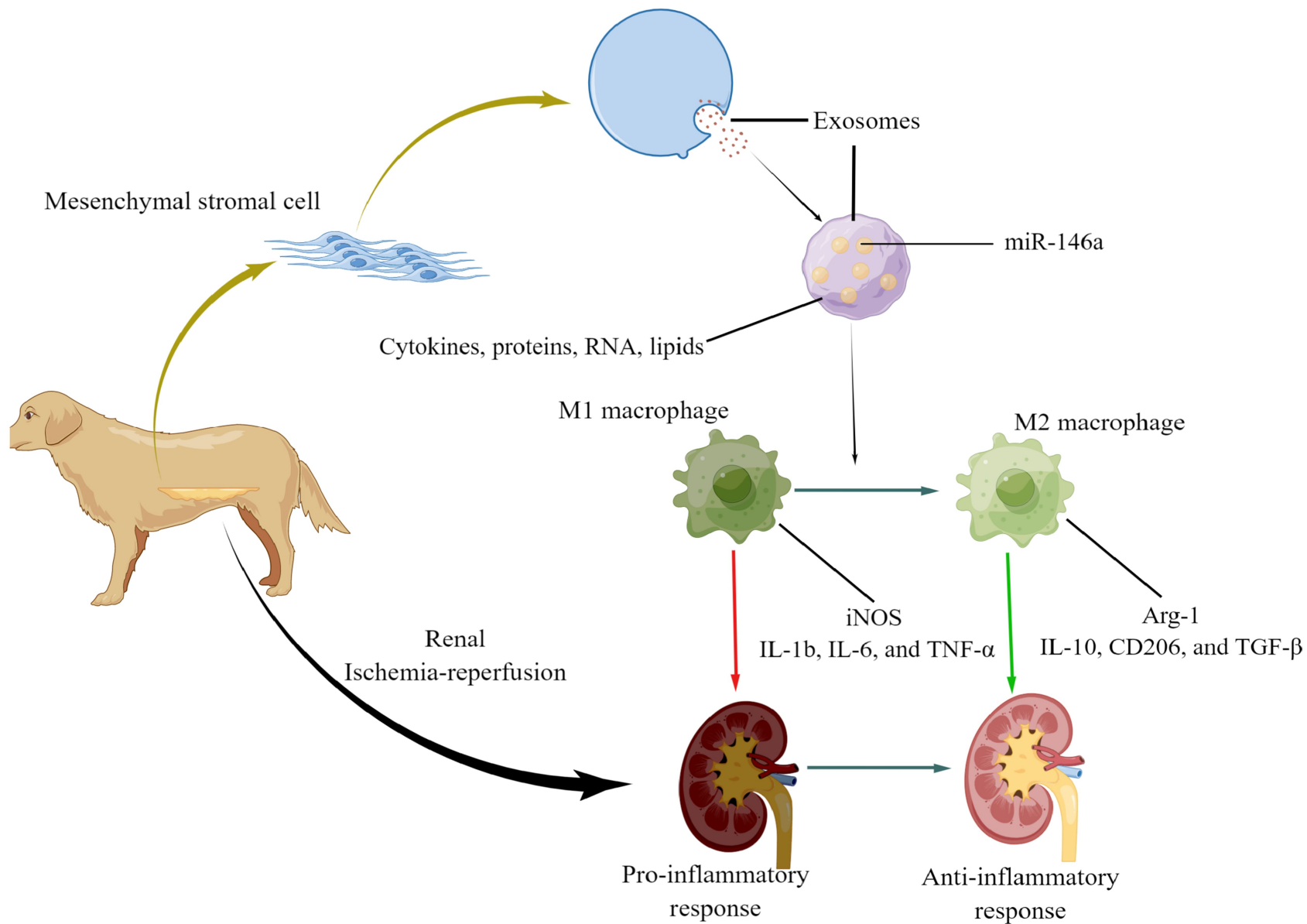

Supplement: Supplementary file 1 [file Data_Sheet_1.PDF]
